# Qishen granules exerts cardioprotective effects on rats with heart failure via regulating fatty acid and glucose metabolism

**DOI:** 10.1186/s13020-020-0299-9

**Published:** 2020-03-04

**Authors:** Kuo Gao, Jian Zhang, Pengrong Gao, Qiyan Wang, Ying Liu, Junjie Liu, Yili Zhang, Yan Li, Hong Chang, Ping Ren, Jinmin Liu, Yong Wang, Wei Wang

**Affiliations:** 1 Dongfang Hospital, Beijing University of Chinese Medicine, Beijing, 100078 China; 2 School of Life Science, Beijing University of Chinese Medicine, Beijing, 100029 China; 3 School of Traditional Chinese Medicine, Beijing University of Chinese Medicine, Beijing, 100029 China; 4 Traditional Chinese Medicine College, North China University of Science and Technology, Tangshan, 063210 Hebei China; 5 School of Chinese Materia Medica, Beijing University of Chinese Medicine, Beijing, 100029 China

**Keywords:** Cardiac functions, Fatty acid metabolism, Glucose oxidation, Glycolysis, Heart failure, Myocardial energy metabolism

## Abstract

**Background:**

*Qishen* granules (QSG) has been applied to treat heart failure (HF) for decades. Our previous transcriptomics study has suggested that *Qishen* granules (QSG) could regulate the pathways of cardiac energy metabolism in HF, but the specific regulatory mechanism has not yet been clarified. This study was to investigate the potential mechanism of QSG in regulating myocardial fatty acid (FA) and glucose metabolism in a rat model of HF.

**Methods:**

The model of HF was induced by left anterior descending coronary artery ligation. Cardiac structure and function were assessed by cine magnetic resonance imaging (MRI) and echocardiography. Level of glucose metabolism was non-invasively evaluated by ^18^F-fluorodeoxyglucose positron emission tomography/computed tomography (PET/CT). Blood lipid levels were determined by enzymatic analysis. The mitochondrial ultrastructure was observed with a transmission electron microscope. The critical proteins related to FA metabolism, glucose metabolism and mitochondrial function were measured by western blotting. The ANOVA followed by a Fisher’s LSD test was used for within-group comparisons.

**Results:**

QSG ameliorated cardiac functions and attenuated myocardial remodeling in HF model. The levels of serum TC, TG and LDL-C were significantly reduced by QSG. The proteins mediating FA uptake, transportation into mitochondria and β-oxidation (FAT/CD36, CPT1A, ACADL, ACADM, ACAA2 and SCP2) as well as the upstreaming transcriptional regulators of FA metabolism (PPARα, RXRα, RXRβ and RXRγ) were up-regulated by QSG. As to glucose metabolism, QSG inhibited glycolytic activity by decreasing LDHA, while stimulated glucose oxidation by decreasing PDK4. Furthermore, QSG could facilitate tricarboxylic acid cycle, promote the transportation of ATP from mitochondria to cytoplasm and restore the mitochondrial function by increasing SUCLA2, CKMT2 and PGC-1α and decreasing UCP2 simultaneously.

**Conclusion:**

QSG improved myocardial energy metabolism through increasing FA metabolism,inhibiting uncoupling of glycolysis from glucose oxidation.

## Background

Heart failure (HF) is a major cause of cardiovascular morbidity and mortality. Despite of the great progress in drug and device therapy, the 5-year mortality rate of HF patient remains alarming [1, 2]. There’re more than 37.7 million HF patients worldwide and the total economic burden of HF was estimated at $108 billion per annum [3, 4]. The effect of current clinical drugs, such as β-blockers, angiotensin-converting enzyme inhibitors and aldosterone antagonists, are still unsatisfied due to the potential inhibition on neuroendocrine system [5]. Therefore, new therapeutic strategies for HF are urgently needed.

Energy metabolism has become the promising therapeutic targets for HF [6]. HF is characterized by dysfunction of generating adenosine triphosphate (ATP) to maintain cardiac contractility due to the metabolic imbalance of fatty acid (FA) and glucose metabolisms [7]. In the healthy circumstances, fatty acid oxidation (FAO) is the major resource of ATP [6]. In the failing heart, both FAO and glucose oxidation were inhibited compensated by glycolysis [8, 9]. The enhanced activity of glycolysis uncoupled from glucose oxidation will lead to energy deficiency and lactate accumulation which can eventually deteriorate the HF [10, 11]. FA metabolism is mainly mediated by fatty acid translocase/cluster of differentiation 36 (FAT/CD36)—carnitine palmitoyltransferase 1 (CPT1)—FAO pathway which is transcriptionally regulated by nuclear factor peroxisome proliferator activated receptor alpha (PPARα)—retinoid x receptors (RXRs) axis [12]. The glucose metabolic changes are accompanied by alterations in lactate dehydrogenase A (LDHA) and pyruvate dehydrogenase kinase 4 (PDK4). At present, it’s considered to be effective therapeutic strategies for HF to promote FA metabolism by facilitating β-oxidation or activating PPARα and enhance glucose oxidation through PDK inhibitor [9].

Traditional Chinese medicine (TCM) has been approved as an attractive candidate for promoting energy metabolism in HF therapy due to its multi-components and multitargets characteristics. *Qishen* granules (QSG) is composed of Radix Astragali, Radix salvia miltiorrhizae, Flos Lonicerae, Radix Scrophulariae, Radix Aconiti Lateralis Preparata, Radix Glycyrrhizae (Table 1) and the ratio of these herbs was 30:15:10:10:9:6. QSG is a frequently prescribed formula with impressive cardio-protective properties for many years in China [13]. Previous studies reported that QSG could improve microcirculation by exerting anti-inflammatory, anti-apoptosis and anti-fibrosis effects [14–19]. In our previous study, mRNA transcriptomic analysis was used to investigate the regulatory pathway of QSG on HF rat model [20]. Interestingly, transcriptomic analysis results indicated QSG could prevent HF by regulating FA and glucose metabolism [20], but the specific regulatory mechanism on FA and glucose metabolism in the treatment of HF has not been investigated so far. In the current study, the critical molecules related to FA metabolism, glucose metabolism and mitochondrial function were measured in a rat model of HF induced by acute myocardial infarction (AMI) to explore the protective effects of QSG on HF.

**Table 1:** Pharmaceutical ingredients of *Qishen* granule

Latin name	Species	Family	Part used
Radix Astragali	*Astragalus membranaceus* (Fisch.) Bge.var. *Mongholicus* (Bge.) Hsiao	*Leguminosae*	Roots
Radix salvia miltiorrhizae	*Salvia miltiorrhiza* Bge.	*Labiatae*	Roots
Flos Lonicerae	*Lonicera japonica* Thunb. *Lonicera confusa* DC. *Lonicera hypoglauca* Miq. *Lonicera dasystyla* Rehd.	*Capridedefoliaceae*	Flowers
Radix Scrophulariae	*Scrophularia ningpoensis* Hemsl.	*Scrophulariaceae*	Roots
Radix Aconiti Lateralis Preparata	*Aconitum carmichaeli* Debx.	*Ranunculaceae*	Roots
Radix Glycyrrhizae	*Glycyrrhiza uralensis* Fisch.	*Leguminosae*	Roots

The ratio of these herbs was 30:15:10:10:9:6

## Materials and methods

### Experimental animals

Male Sprague–Dawley rats with weights of 240 ± 10 g were obtained from the Vital River Laboratory Animal Technology Co. Ltd. (Beijing, China). The room in which rats were housed was 23 ± 2 °C and 55 ± 5% relative humidity with artificial 12:12 h equivalent light–dark cycles. All experimental procedures were conducted according to the National Institute of Health Guide for the Care and Use of Laboratory Animals, and approved by the Animal Care Committee of Beijing University of Chinese Medicine.

### Preparation and quantitative analysis of QSG

QSG consists of 6 Chinese herbs, including Radix Astragali, Radix salvia miltiorrhizae, Flos Lonicerae, Radix Scrophulariae, Radix Aconiti Lateralis Preparata, Radix Glycyrrhizae. The Chinese herbs were identified by Professor Dr. Jian Ni, School of Chinese Materia Medica, Beijing University of Chinese Medicine. The voucher specimens (Voucher numbers: HQ-2016-007; DQ-2016-008; JYH-2016-009; XS-2016-010; FZ-2016-011; GC-2016-012) were submitted to Department of Chinese medicine teaching and Research, School of Traditional Chinese Medicine, Beijing University of Chinese Medicine. The drug used in this study was the same batch as the previously published study [16]. The fingerprint spectrum was established by the high performance liquid chromatography method to control the quality of the QSG. The results of the quality control of QSG were described in detail in our previous study [16].

### Animal grouping, HF model induction and drug administration

Total 60 rats were randomly divided into 4 groups (15 rats per group): sham, model, QSG and fosinopril groups. Rats were anesthetized with 1% pentobarbital sodium (45 mg/kg) by intraperitoneal injection before received ligation surgery of left anterior descending (LAD) coronary artery as previously reported [14]. Briefly, left thoracotomy between third and fourth intercostal space was performed on rats. After exposing the cardiac tissues, LAD was ligated with a sterile suture (Shuangjian, Shanghai, P. R. China) 1 mm below the left atrium. The thorax was then closed layer by layer. After thoracotomy, rats were warmed on a heated blanket. Sham-operated rats were manipulated in the same way, with no actual ligation of LAD. There was no animal death in the sham group during the entire experiment, while the mortality rate of rats in the model group, QSG group and fosinopril group was 26.7%, 13.3%, and 13.3% during the entire experiment, respectively.

QSG and fosinopril (Bristol-Myers Squibb, China) were dissolved in sterile saline. The rats in the QSG group were treated with QSG at a daily dose of 18.66 g/kg for 28 days as previous study [16]. The rats in the positive control group were treated with fosinopril at a daily dose of 1.2 mg/kg as previous study [16]. Rats in the sham group and model group were administered with normal saline (10 ml/kg/day).

### Assessment of cardiac functions by echocardiography

Echocardiography was applied to detect the Left ventricular end-diastolic diameter (LVEDD), left ventricular end-systolic diameter (LVESD), ejection fraction (EF), fractional shortening (FS). A PST 65A sector scanner (8-MHz probe) was employed, which generates two-dimensional images at a frame rate of 300 to 500 frames/s. The left ventricular dimension was measured using M-model ractional shortening, and FS was calculated using the following equation:

$${\text{FS }} = \, \left[ {\left( {{\text{LVEDD }} - {\text{ LVESD}}} \right)/{\text{ LVEDD}}} \right] \, \times { 1}00\% .$$

EF was calculated using the following equation:

$${\text{EF }} = \, \left[ {\left( {{\text{LVEDV }} - {\text{ LVESV}}} \right)/{\text{LVEDV}}} \right] \, \times { 1}00\% .$$

### Assessment of cardiac functions and myocardial remodeling by cine MRI

Rats (n = 6 per group) were inhaled anesthetized with 1.5–2% isoflurane by isoflurane anesthesia system (JD Medical Dist. Co. Inc., USA) before scanning. Minor adjustments of anesthesia would be made so that heart rate could be kept as constant as possible during the experiment, considering the effect of heart rate on imaging. After the rats were fully anesthetized, the animal was moved to a 7-T MRI (Bruker pharmascan) scanner and placed in a supine position on a scan bed. A standard echo axis was first performed on a gradient echo scan of a rat thoracic cavity, followed by a cardiac movie scan. Transverse magnetic resonance images were obtained for the body region containing the heart. 11 to 12 layers of ventricular short-axis movie images were performed at the end of expiration. MR images were acquired using a flash sequence with 2.5 ms of echo time, 10 ms of repetition time, 15° of flip angle, 1 mm of thickness, 0 mm of gap, 60 mm × 60 mm field of view (FOV), 2 of number of excitations, 192 × 192 of matrix, the sequence was ECG-triggered and respiratory gated. 20 frames of movie images were captured during each cardiac cycle.

All images were segmented and calculated by the MRI software VnmrJ 3.1. Regional left ventricular (LV) function and wall motion was visually appreciated on cine MRI and short-axis slices from the base to the apex of the heart were used for quantitative assessment. Myocardium segmentation was performed among at least 8 short-axis slices outlining both, endocardial and epicardial borders in all the cardiac frames. The contour curves of left ventricular end-diastole and left ventricular end-systole were drawn manually at the level of short axis in each slice so that endocardial area could be got by the software. Left ventricular end-diastolic volume (LVEDV) and Left ventricular end-systolic volume (LVESV) were calculated from the largest and smallest areas, respectively, according to the formula [21]:

$$\begin{aligned} {\text{LV volume}} & = \mathop \sum \limits^{all slices} \left( {endocardial\,area} \right) \\ & \quad \times \left( {distance\,between\,slice\,centers} \right). \end{aligned}$$

To minimize measurement errors, apical and basal slices without blood pool at either end-systole or end-diastole were disregarded [22]. Left ventricular ejection fraction (LVEF) was calculated as follow:

$${\text{LVEF}} = \frac{{\left( {{\text{LVEDV}} - {\text{LVESV}}} \right)}}{\text{LVEDV}} \times 100\% .$$

LVEDD, LVESD, left ventricular end-diastolic anterior wall thickness (LVEDAWT), and left ventricular end-systolic anterior wall thickness (LVESAWT) were measured at the level of papillary muscle. Left ventricular fractional shortening (LVFS) was calculated as follow:

$${\text{LVFS}} = \frac{{\left( {{\text{LVEDD}} - {\text{LVESD}}} \right)}}{\text{LVEDD}} \times 100\% .$$

### Assessment of glucose metabolism by PET/CT imaging

Prior to scanning, rats (n = 6 per group) in the sham, model and QSG groups were prohibited from eating for more than 12 h to enhance tracer uptake into the myocardium [23] and then received an injection of ^18^F-FDG (5.5 MBq/kg) via abdominal cavity. Anesthesia was initiated and maintained using the same protocol as MRI examination. A micro PET P4 dedicated animal PET system (Siemens Preclinical Solutions, Knoxville, TN, USA) was used in this study. Briefly, the system has an axial FOV of 7.8 cm and a translational FOV of 12.7 cm, with a spatial resolution of 0.7 mm at the center of the FOV. Prior to imaging, the system was calibrated by imaging a rat-sized cylinder phantom filled with a known concentration of FDG (500 uci). After a low-dose CT scan for attenuation correction (tube voltage 80 kV; tube current 500 ua, total rotation degrees 220, rotation steps 120, exposure time 0.26 s, slice thick 0.2 mm), PET was performed to evaluate myocardial uptake. The heart was made in the center of the FOV, and then PET emission images of the thorax were obtained for 15 min. Data were reconstructed using the iterative OSEM3D/MAP algorithm provided by the system manufacturer, with all available corrections applied, including that for attenuation and scattering. The images were analyzed using the software Inveon Research Workplace (Siemens Preclinical Solutions, USA) with iterative ordered-subsets expectation maximization (2 iterations and 8 subsets). The matrix size was 2.0 × 2.0 mm with 128 × 128 pixels, and the slice thickness was 3.125 mm. In addition, ^18^F-FDG uptake in the regions of interest was also judged quantitatively by measuring the mean standard uptake value (SUV_mean_), the max standard uptake value (SUV_max_), and the min standard uptake value (SUV_min_) of LV and global heart.

### Measurement of lipid metabolism in serum

Rats were anesthetized with 1% pentobarbital sodium (50 mg/kg) by intraperitoneal injection, and then blood samples (n = 6 per group) were collected from the abdominal aorta and centrifuged at 1000×*g* for 20 min to obtain serum. Serum total cholesterol (TC), triglyceride (TG), low-density lipoprotein cholesterol (LDL-C) and high-density lipoprotein cholesterol (HDL-C) levels were measured by automatic biochemical analyzer (HITACH17080, Tokyo, Japan) following the instructions of kits (Sekisui chemical company, Tokyo, Japan).

### Hematoxylin–eosin staining

The hearts (n = 4) were excised and irrigated with saline solution. Four left ventricles in each group were fixed in 4% paraformaldehyde solution for more than 48 h and embedded in paraffin. Sections (5 μm thick) were cut for further histological analysis. Hematoxylin–eosin (HE) staining was performed to visualize cardiomyocyte architecture [24]. Images were visualized under an optical microscope at 400× magnification.

### Mitochondrial ultrastructure observation using a transmission electron microscope

The 1 mm × 1 mm × 2 mm cardiac tissues of left ventricle in infarct border zone (n = 4) which were obtained from each group randomly were fixed in 4% glutaraldehyde more than 2 h, in 1% osmic acid for 1–2 h, and then washed by PBS buffer solution 5 min (3 times). And after dehydration, permeation, embedding, and ultrathin sections cut. Ultrastructural alterations in heart tissues were observed using a transmission electron microscope (Hitachi, Tokyo, Japan).

### Measurement of lactate in serum and myocardial Tissue

Lactate in serum and myocardial tissue was determined by lactic acid assay kit (Nanjing Jiancheng Bioengineering institute, A019-2-1).

### Western blotting

Cardiac tissues of left ventricle in infarct border zone were rapidly frozen in nitrogen and stored at − 80 °C for further experiments. Western blotting was performed as previous study [14]. Briefly, proteins were extracted from cardiac tissues, using RIPA buffer (50 mM Tris–HCl PH7.4, 150 mM NaCl, 1% NP-40 and 0.1% SDS containing a protease inhibitor cocktail (Sigma, St. louis, MO, USA). Equal amounts of protein were subjected to sodium dodecyl sulfate polyacrylamide gel electrophoresis (SDS-PAGE) and transferred onto polyvinylidene fluoride membranes. Standard western blot analysis was conducted using FAT/CD36 (1:500 dilutio, Abcam: ab64014). Glyceraldehyde 3-phosphate dehydrogenase (GAPDH) antibody (1:10,000 dilution, Cell Signaling Technology: 5174s) was used as a loading control. After incubation with the appropriate secondary antibodies, signals were visualized using the ECL Plus Western blotting detection reagents (Bio-Rad) for 1 min at room temperature. The bands in the membrane were visualized and densitometric analysis of band intensity was performed using Imagelab software (Bio-Rad, Hercules, CA, USA). Then the same procedure was taken to detect the CPT1A (1:2000 dilution; Abcam: ab128568), acetyl-coenzyme a acyltransferase 2 (ACAA2) (1:2000 dilution; Abcam: ab128911), acyl-coa dehydrogenase long chain (ACADL) (1:3000 dilution; Abcam: ab196655), acyl-coA dehydrogenase medium chain (ACADM) (1:4000 dilution; Abcam: ab110296), sterol carrier protein 2 (SCP2) (1:3000 dilution; Abcam: ab140126), PPARα (1:2000 dilution; Abcam: ab8934), retinoid x receptor α (RXRα) (1:4000 dilution; Abcam: ab125001), retinoid x receptor β (RXRβ) (1:2000 dilution; Cell Signaling Technology: 8715s), retinoid x receptor γ (RXRγ, 1:3000 dilution; Abcam: ab15518), succinate-coa ligase ADP-forming β-subunit 2 (SUCLA2) (1:2000 dilution; Abcam: ab202582), creatine kinase mitochondrial 2 (CKMT2) (1:2000 dilution; Abcam: ab55963), peroxisome proliferator-activated receptor gamma coactivator 1α (PGC-1α) (1:2000 dilution; Abcam: ab54481), uncoupling protein 2 (UCP2) (1:4000 dilution, Cell Signaling Technology: 89326 s), LDHA (1:500 dilution; Abcam: ab101562) and PDK4 (1:1000 dilution; Abcam: ab214938).

### Statistics analysis

Data are expressed as mean ± standard error (SE). ANOVA using SPSS 17.0 software (SPSS, Chicago, IL, USA) was applied to evaluate between-group differences in the outcome variables, follow-up least significant differences (LSD) analysis verified these differences were significant. A significant difference was considered if the *p* value was less than 0.05.

## Results

### Effects of QSG on cardiac functions and myocardial remodeling in HF rats after AMI

**Fig. 1:**
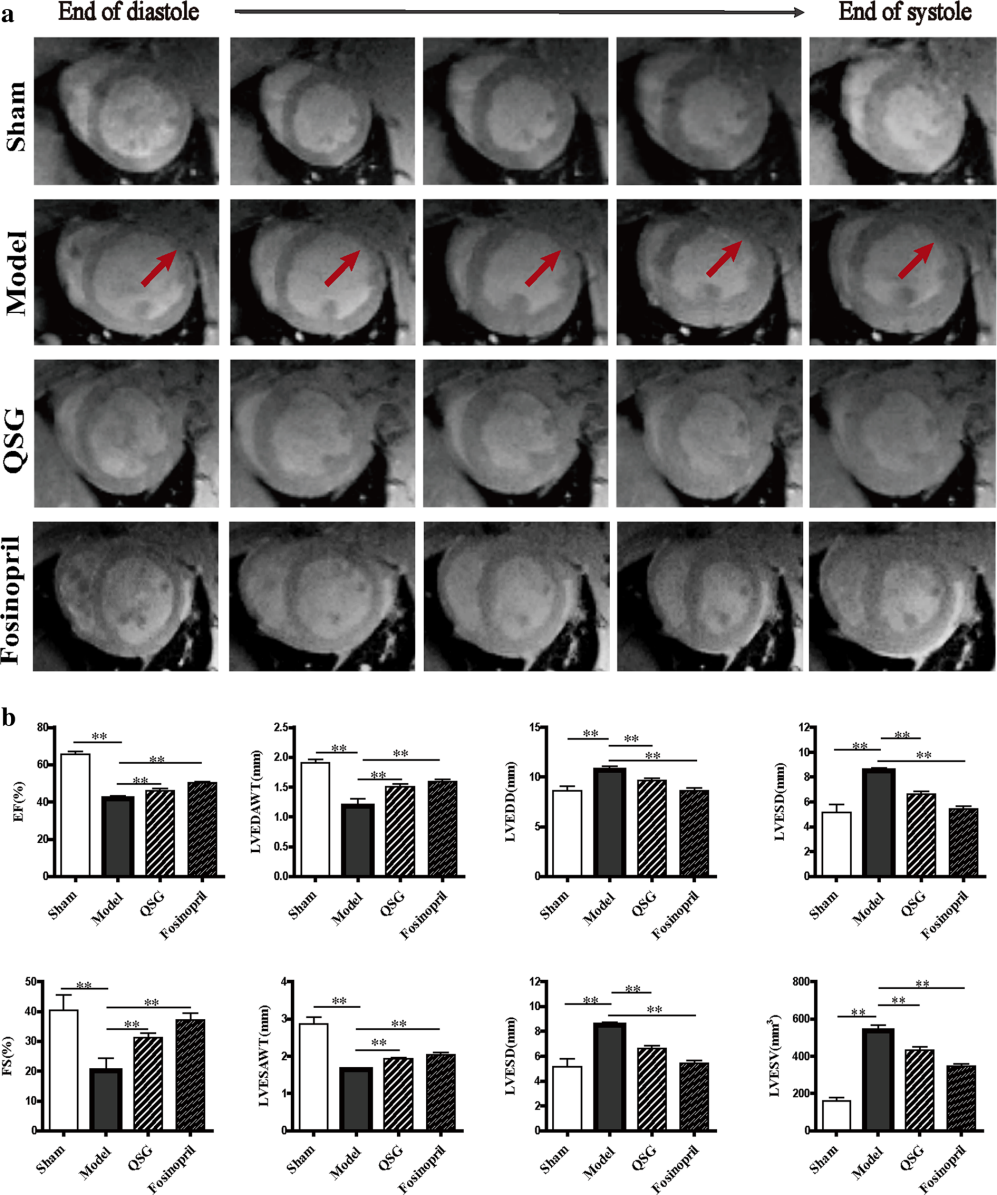
Effects of QSG on cardiac structure and function by MRI in HF rats after AMI. **a** Representative MR images of rat hearts at the slice of papillary muscle in short-axis view in the four groups. From left to right was the process from the end of diastole to the end of systole. The anterior wall of the left ventricle in the model group was thinner (red arrow) while ventricular cavity was larger in the model group. After drug treatment, ventricular remodeling was attenuated in QSG and fosinopril group. **b** Bar graphs of LV parameters in the four groups. Rats in the model group underwent significant drops in EF, FS, LVEDAWT and LVESAWT and increases in LVEDD, LVESD, LVEDV, LVESV compared to the sham group. LVEDD, LVESD, LVEDV and LVESV decreased observably, meanwhile LVEDAWT, LVESAWT, FS and EF increased obviously in QSG and fosinopril group compared with the model group. n = 6 per group. Values are mean ± SE. Asterisks indicates significant differences. **P < 0.01

MRI results showed that the anterior wall of the left ventricle in HF model rats was thinner and ventricular cavity was larger than those in sham-operated rats (Fig. 1). After treatment for 28 days, ventricular remodeling was attenuated in the QSG and fosinopril groups (Fig. 1). Left ventricle parameters, including LVEDAWT, LVESAWT, LVEDD, LVESD, LVEDV, LVESV, EF and FS, in each group were detailed in Fig. 1. LVEDAWT and LVESAWT were decreased whereas LVEDD, LVESD, LVEDV and LVESV were increased in the model group as compared to the sham group (*P *< 0.01, Fig. 1). The cardiac functions were impaired in the model group, as manifested by significant reduction in FS and EF (*P *< 0.01 versus the sham group, Fig. 1). The results were consistent with MRI images (Fig. 1). QSG and fosinopril could restore the LVEDD, LVESD, LVEDV and LVESV, and increase the LVEDAWT, LVESAWT, FS and EF significantly compared to the model group (*P *< 0.01, Fig. 1), suggesting a definite cardioprotective effect of QSG on HF.

Echocardiography showed down-regulation of EF and FS in the model group as compared with the sham group (*P* < 0.01, Table 2). This was accompanied by the enlargement of left ventricular end-diastolic diameter (LVEDD) and left ventricular end-systolic diameter (LVESD) (*P* < 0.01, Table 2). In contrast, EF and FS increased obviously while LVEDD and LVESD decreased significantly in the QSG and fosinopril groups compared to the model group (*P* < 0.01 or *P* < 0.05, Table 2), suggesting QSG could improve cardiac functions in HF.

**Table 2:** Assessments of cardiac function by echocardiography

Group	EF (%)	FS (%)	LVEDD (mm)	LVESD (mm)
Sham	89.03 **± **1.27**	61.02 **± **1.87**	6.62 ± 0.15**	3.15 ± 0.22**
Model	34.60 **± **3.46	17.59 **± **1.90	9.81 ± 0.19	8.14 ± 0.44
QSG	49.11 **± **5.16*	26.60 **± **3.44*	8.11 ± 0.26*	6.51 ± 0.49*
Fosinopril	52.61 **± **4.38**	28.90 **± **2.98**	8.92 ± 0.60*	6.55 ± 0.59*

n = 6 per group. Values are represented as mean ± SE. Statistically different from the model group: *P < 0.05, **P < 0.01

### Effects of QSG on blood lipid indicators in HF rats after AMI

**Fig. 2:**
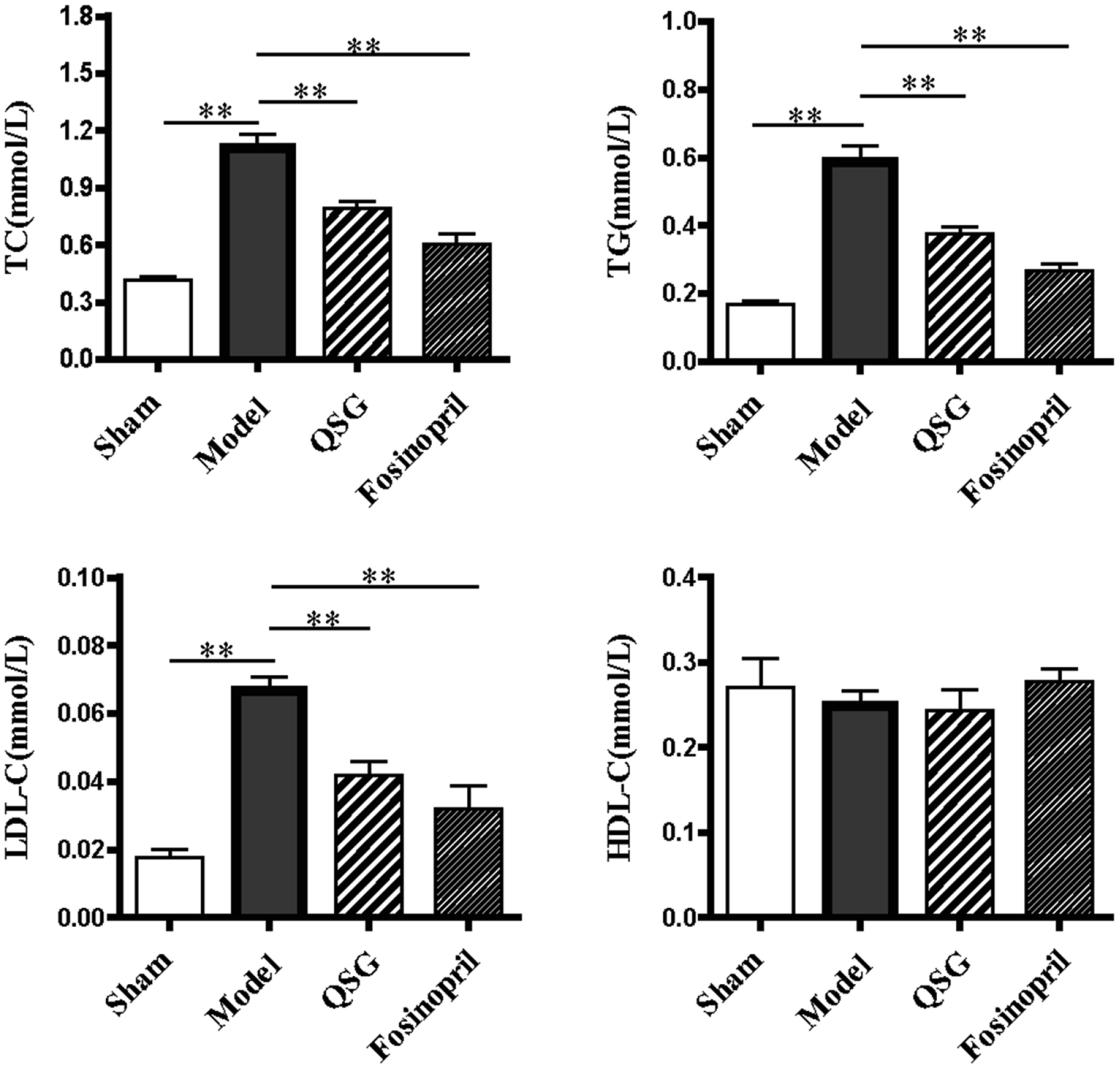
Effects of QSG on blood lipid indicators in HF rats after AMI. Serum TC, TG, and LDL-C levels in the model group were up-regulated compared with sham group. After treatment with QSG and Fosinopril, TC, TG, and LDL-C levels were reduced. HDL-C showed no significant change in the four groups. n = 6 per group. Values are mean ± SE. Asterisks indicates significant differences. **P < 0.01

TC, TG and LDL-C levels are regarded as the independent risk factors of HF. Enzymatic analysis indicated that TC, TG, and LDL-C levels in the model group were up-regulated compared with the sham group (*P* < 0.01, Fig. 2). After treatment with QSG and Fosinopril, TG, TC and LDL-C levels were significantly reduced (*P* < 0.01, Fig. 2). HDL-C showed no significant changes among the different groups (*P* > 0.05, Fig. 2).

### Effects of QSG on myocardial morphological injury in HF rats after AMI

**Fig. 3:**
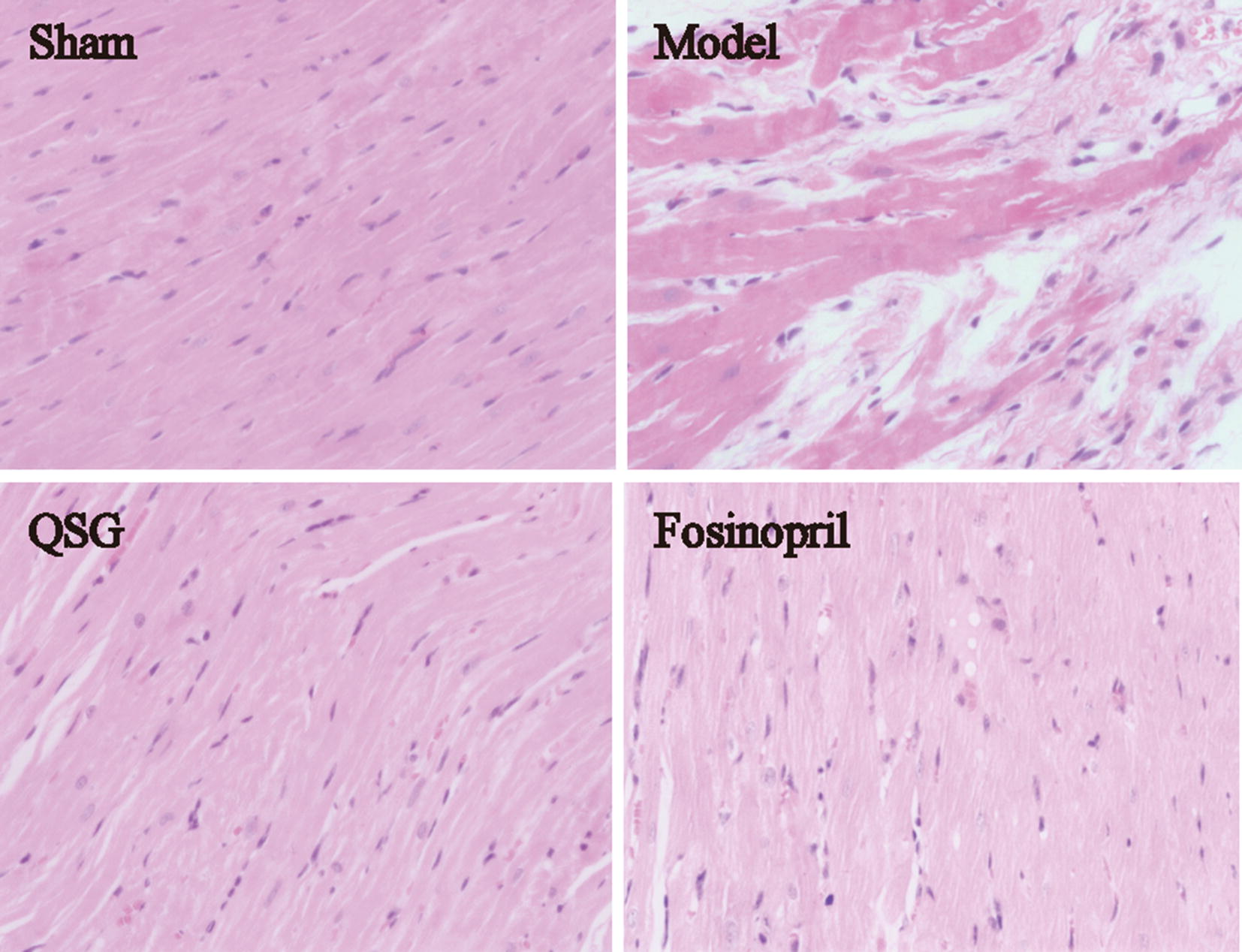
Effects of QSG on myocardial morphological injury in HF rats after AMI. Myocardial samples of the four groups surrounding an area of infarction, visualized via hematoxylin–eosin staining (400×). The cardiomyocytes in the sham group were orderly arranged and the nuclei were lightly stained. The myocardial tissue in the model group exhibited obvious pathological abnormalities with pyknotic dark-staining nuclei and inflammatory cell infiltration. QSG and fosinopril could attenuate cellular degeneration and inflammatory cell infiltration

Results of HE staining demonstrated that cardiomyocytes in the sham group were orderly arranged and the nuclei were lightly stained (Fig. 3). The myocardial tissue in the model group exhibited obvious pathological abnormalities with pyknotic dark-staining nuclei and inflammatory cell infiltration. QSG and fosinopril could attenuate cellular degeneration and inflammatory cell infiltration (Fig. 3).

### Effects of QSG on FAT/CD36-CPT1-FAO pathway in HF rats after AMI

**Fig. 4:**
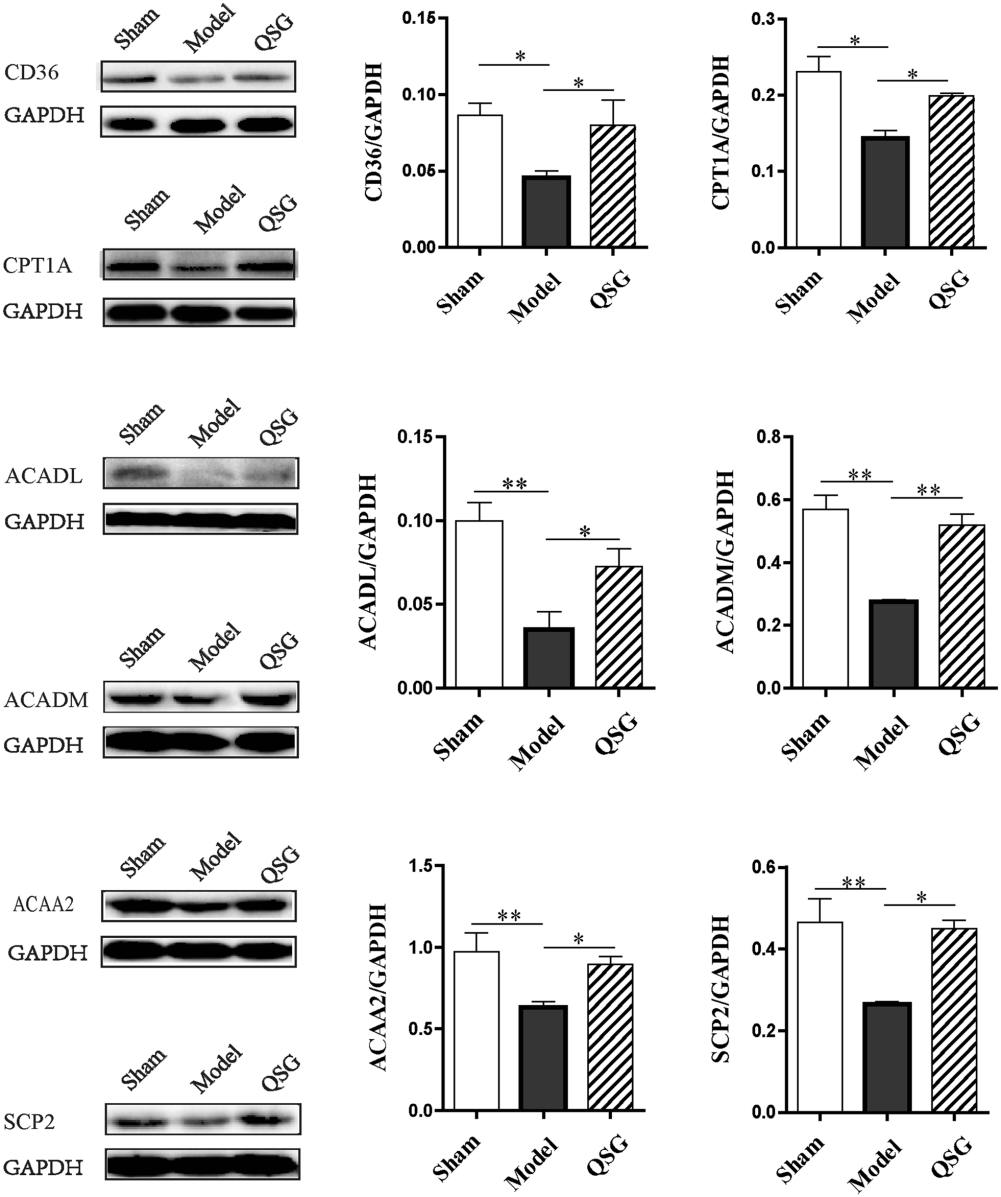
Effects of QSG on FAT/CD36-CPT1-FAO pathway in HF rats after AMI. The protein expressional levels of CD36, CPT1A, ACADL, ACADM, ACAA2 and SCP2 in the three groups were significantly decreased in the model group compared with the sham group. After treatment with QSG, cardiac CD36, CPT1A, ACADL, ACADM, ACAA2 and SCP2 levels were all significantly increased. n = 4 per group. Values are mean ± SE. Asterisks indicates significant differences. *P < 0.05, **P < 0.01

Since QSG could regulate the blood lipid as showed above, we further detected the uptake, transportation and β-oxidation of FA which mainly mediated by FAT/CD36-CPT1-FAO pathway in myocardiocytes [9]. FAT/CD36 medicates the entry of long-chain FA into cardiac cells [25], and CPT1 is the rate-limiting enzyme for transportation of FA from cytoplasm to mitochondria [9]. ACADL, ACADM, ACAA2 and SCP2 facilitate the metabolism of FA through β-oxidation in mitochondria [26–28]. The protein levels of cardiac FAT/CD36, CPT1A, ACADL, ACADM, ACAA2 and SCP2 were all impressively reduced in the model group compared to the sham group (*P* < 0.05 or *P* < 0.01, Fig. 4), suggesting that the capacity of FA intake, transportation and β-oxidation was severely impaired. Intriguingly, QSG could remarkably up-regulated those indicators (*P* < 0.05 or *P* < 0.01, Fig. 4), indicating that QSG could promote FA metabolism to exert the protective effect on HF.

### Effects of QSG on PPARα-RXRs pathway in HF rats after AMI

**Fig. 5:**
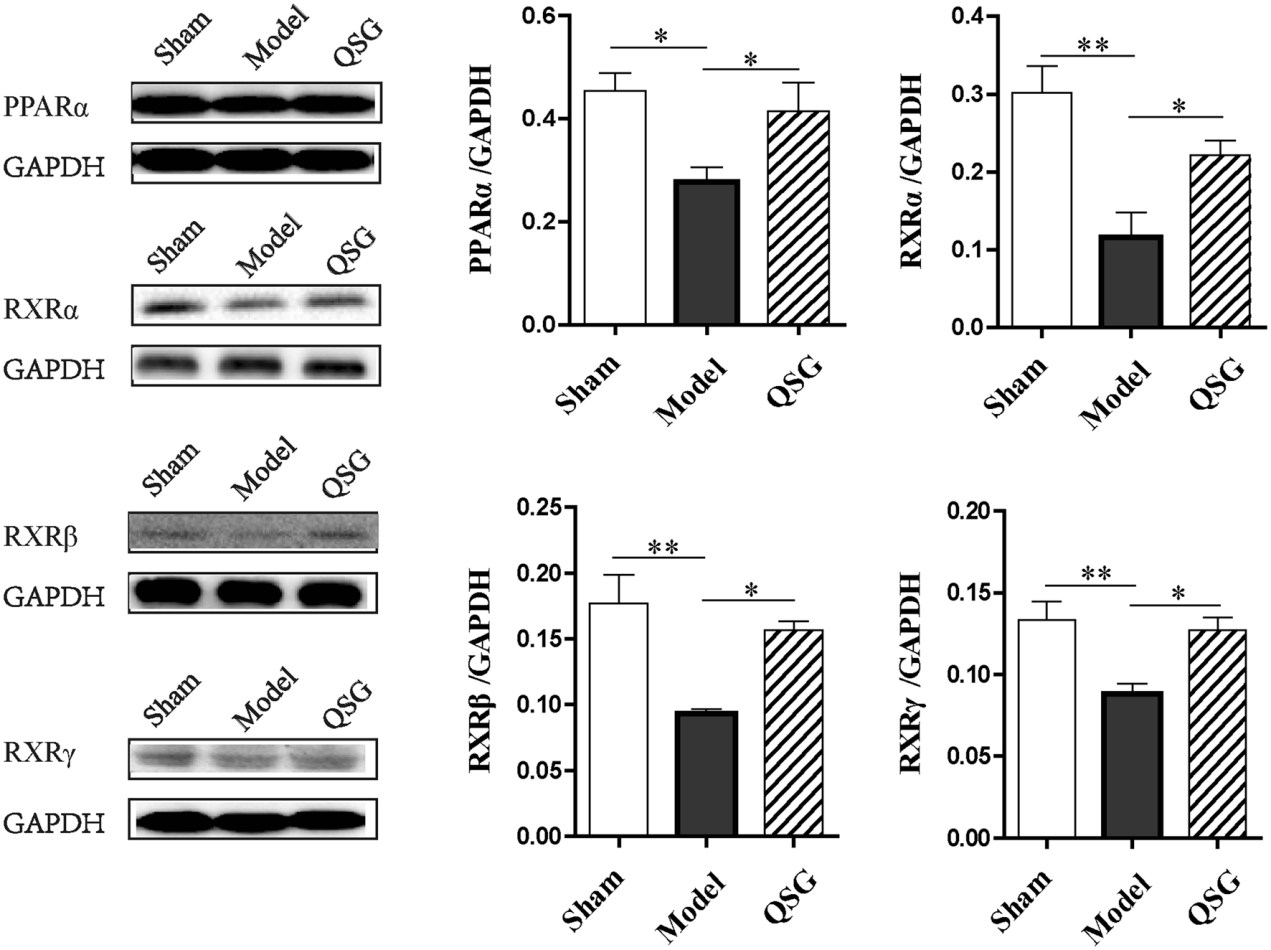
Effects of QSG on PPARα-RXRs pathway in HF rats after AMI. The expression of PPARα, RXRα, RXRβ and RXRγ in protein levels were down-regulated in the sham group compared with the model group. After treatment with QSG, PPARα-RXRs pathway was activated, illustrated by an increased levels of PPARα, RXRα, RXRβ and RXRγ. n = 4 per group. Values are mean ± SE. Asterisks indicates significant differences. *P < 0.05, **P < 0.01

Transcription of the key molecules mentioned above are mainly regulated by PPARα-RXRs pathway in cardiac cells [12]. In the model group, the proteins levels of PPARα, RXRα, RXRβ and RXRγ decreased compared with the sham group (*P* < 0.05 or *P* < 0.01, Fig. 5). After treatment with QSG, PPARα-RXRs pathway was activated, as illustrated by the increased levels of PPARα, RXRα, RXRβ and RXRγ (*P* < 0.05, Fig. 5).

### Effects of QSG on regulating glucose metabolism in HF rats after AMI

**Fig. 6:**
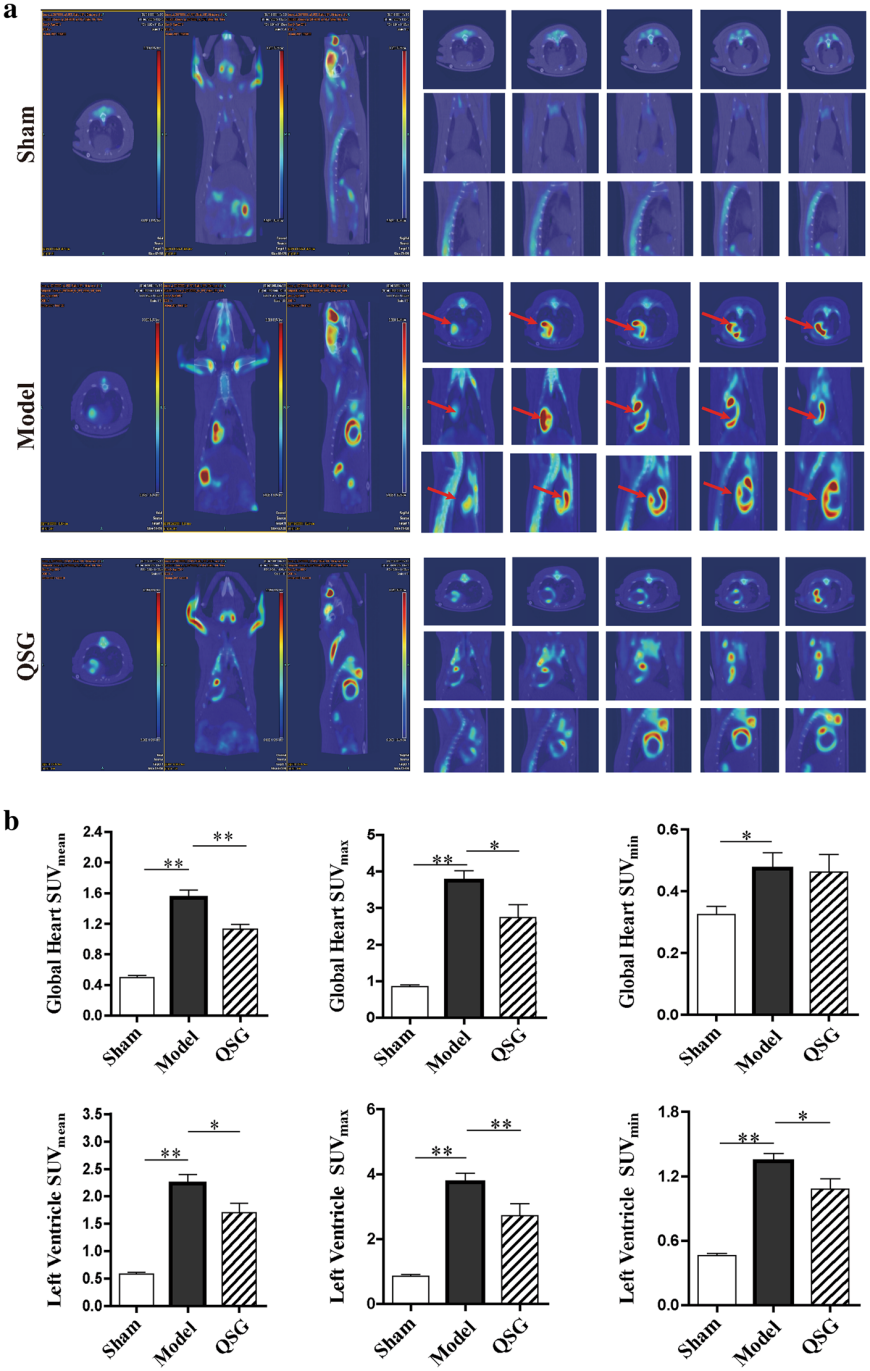
Effects of QSG on regulating glucose metabolism by ^18^F-fluorodeoxyglucose PET/CT in HF rats after AMI. **a** Representative images of different slices of rat hearts were shown in transaxial, coronal and sagittal sections. Abnormally high signals marked with red arrows was noticeable in the model group, indicating that glucose was accumulated abnormally in periinfarct area in HF model rats. The accumulating glucose was significantly metabolized with QSG treatment evidenced by the weakened abnormal signals. **b** Statistical analysis of SUV achieved from ^18^F-FDG PET scans in the three groups. SUV_mean_, SUV_max_, and SUV_min_ of LV and global heart in the model group were significantly higher than sham group. QSG could restore abnormal metabolism by reducing SUV_mean_, SUV_max_, and SUV_min_ of LV and SUV_mean_ and SUV_max_ of global heart. n = 6 per group. Values are mean ± SE. Asterisks indicates significant differences. *P < 0.05, **P < 0.01

Glucose metabolism was assessed non-invasively by ^18^F-FDG PET/CT. PET images displayed transaxial, coronal and sagittal sections of ^18^F-FDG uptake in the different groups (Fig. 6a). Compared with the sham group, ^18^F-FDG uptake of the model group was significantly elevated, as evidenced by the highlight signal shown on PET images (Fig. 6a). Correspondingly, SUV_mean_, SUV_max_, and SUV_min_ of LV and global heart in the model group were remarkably higher than the sham group (*P* < 0.05 or *P* < 0.01, Fig. 6b). QSG could restore abnormal metabolism evidenced by the weakened abnormal signals (Fig. 6a). Correspondingly, SUV_mean_, SUV_max_, and SUV_min_ of LV and SUV_mean_ and SUV_max_ of global heart were significantly decreased in the QSG group (*P* < 0.05 or *P* < 0.01, versus the model group, Fig. 6b).

### Effects of QSG on the uncoupling of glycolysis from glucose oxidation in HF rats after AMI

**Fig. 7:**
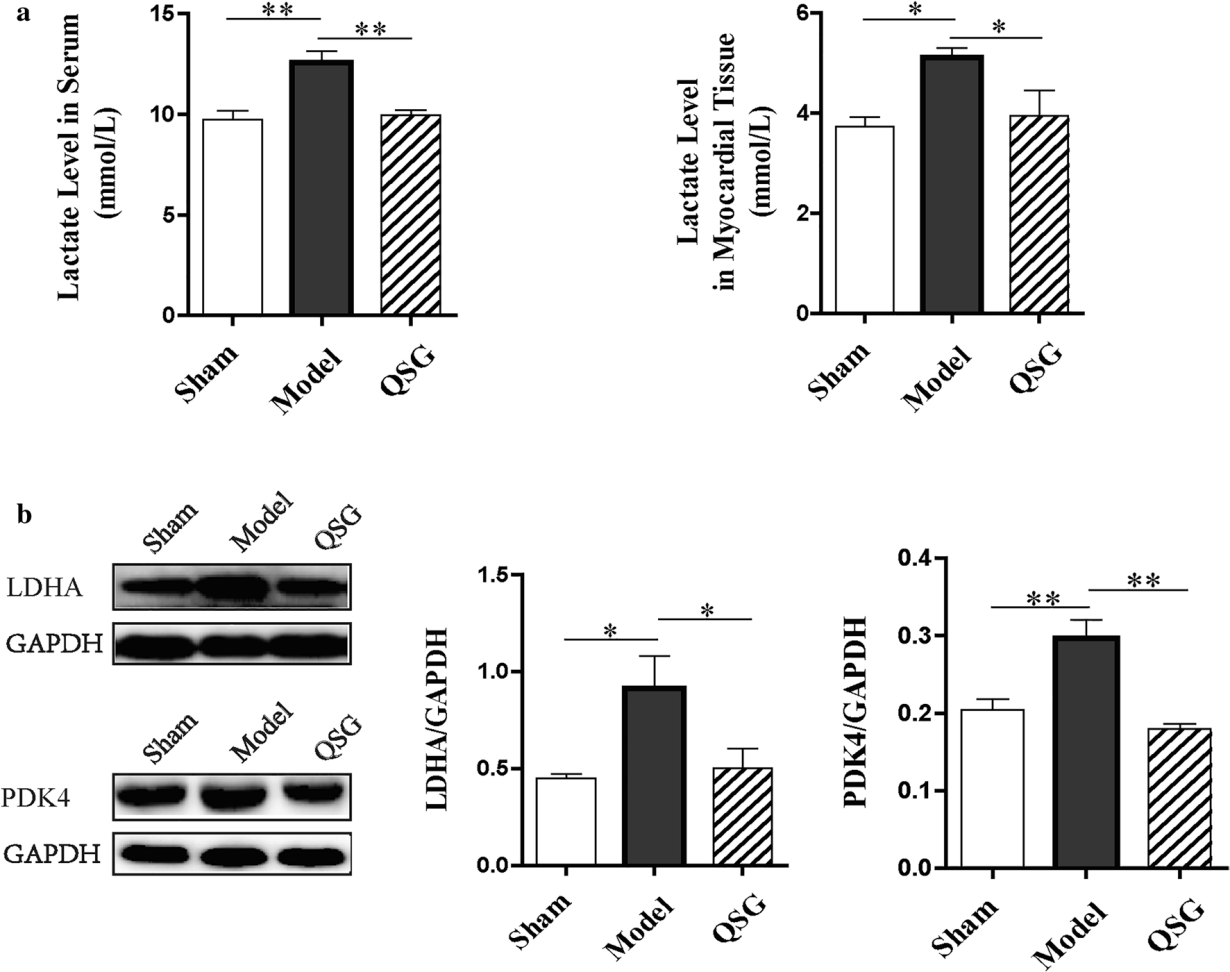
Effects of QSG on the uncoupling of glycolysis from glucose oxidation in HF rats after AMI. **a** Lactate level in serum and myocardial tissue. Lactate level in serum and myocardial tissue was significantly increased following HF and downregulated with QSG treatment. n = 8 per group. Values are mean ± SE. Asterisks indicates significant differences. *P < 0.05, **P < 0.01. **b** Myocardial protein expressional levels of LDHA and PDK4 in the three groups. LDHA involved in regulating glycolysis was observably elevated in the model group. LDHA level was significantly down-regulated by treatment of QSG compared with the model group. PDK4, decreasing glucose oxidation by an inhibitory phosphorylation of the pyruvate dehydrogenase complex, was up-regulated in the model group and down-regulated in the QSG group. n = 4 per group. Values are mean ± SE. Asterisks indicates significant differences. *P < 0.05, **P < 0.01

The level of lactate in serum and myocardial tissue in the model group elevated significantly (*P* < 0.01 or *P* < 0.05, Fig. 7) compared with the sham group. QSG treatment reduced lactate level significantly compared with the model group (*P* < 0.01 or *P* < 0.05, Fig. 7). Cardiac LDHA involved in glycolysis was dramatically elevated in the HF model group (*P* < 0.05, Fig. 7) due to the lack of oxygen in heart. In contrast, expression of LDHA was significantly downregulated in QSG group (*P* < 0.01, Fig. 7). PDK4 decreases glucose oxidation by phosphorylating the pyruvate dehydrogenase complex [29]. Expression of PDK4 was up-regulated in the model group compared with the sham group (*P* < 0.01, Fig. 7) and down-regulated in the QSG group versus the model group (*P* < 0.01, Fig. 7), indicating that QSG inhibited uncoupling of glycolysis from glucose oxidation.

### Effects of QSG on tricarboxylic acid cycle and the transfer of ATP from mitochondria to cytoplasm in HF rats after AMI

**Fig. 8:**
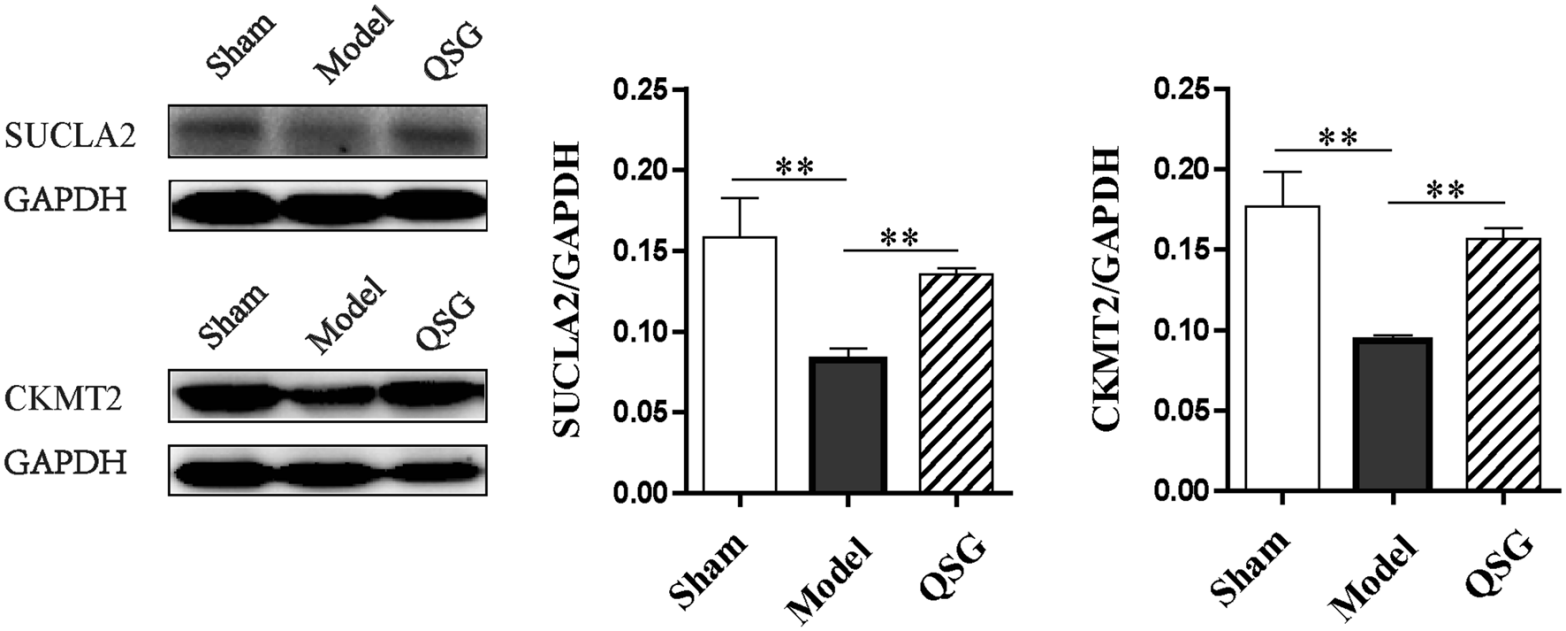
Effects of QSG on TAC and the transfer of ATP from mitochondria to cytoplasm in HF rats after AMI. SUCLA2 is a core enzyme of TAC and CKMT2 is responsible for the transfer of mitochondrial ATP to the cytoplasm. A reduction of SUCLA2 and CKMT2 were observed in the model group when compared to the sham group. QSG increased cardiac CKMT2 and SUCLA2 levels compared with the model group. n = 4 per group. Values are mean ± SE. Asterisks indicates significant differences. *P < 0.05, **P < 0.01

SUCLA2 is a core enzyme of tricarboxylic acid cycle (TAC), participates in acetyl-CoA oxidation [42]; CKMT2, a member of creatine kinase isoenzyme family, is responsible for the transfer of mitochondrial ATP to the cytoplasm [30]. Reduction of SUCLA2 and CKMT2 were observed in HF model group when compared to the sham group (*P* < 0.01, Fig. 8). QSG increased cardiac CKMT2 and SUCLA2 levels (*P* < 0.01 versus the model group, Fig. 8) to facilitate TAC and energy transfer from mitochondria to cytoplasm.

### Effects of QSG on the structure and function of mitochondria in myocardial tissue of HF rats after AMI

**Fig. 9:**
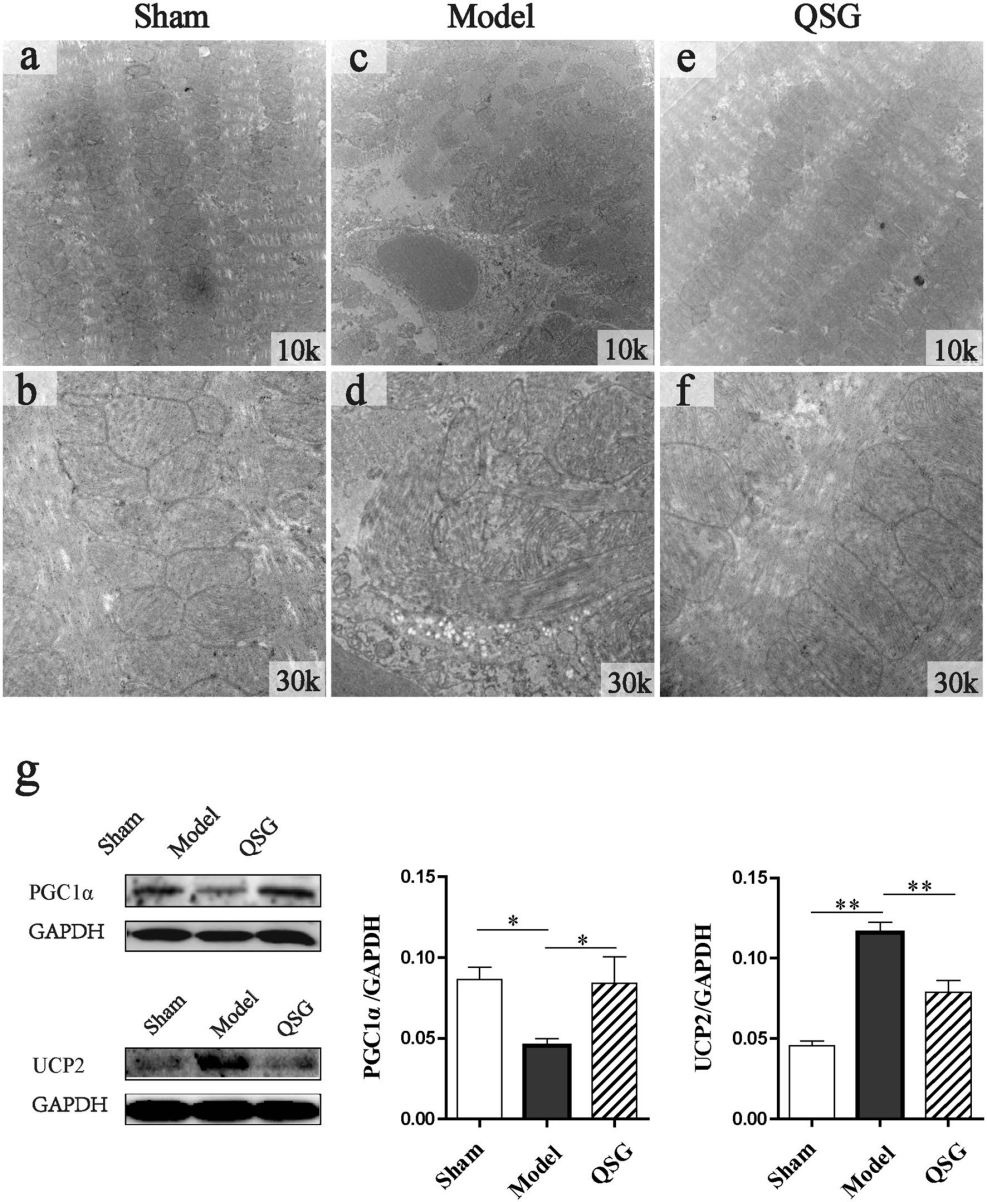
Effects of QSG on the structure and function of mitochondria in myocardial tissue of HF rats after AMI. **a** The mitochondria arranged orderly and densely, and were round or oval in the sham group. **b** The mitochondrial membrane’s structure was complete, and the mitochondrial cristae and matrix are clearly visible and evenly arranged in the sham group. **c**, **d** The mitochondrial arrangement was scattered, swelling was obvious, matrix was loose, and cristae was fuzzy with obvious partial fracture in the model group. **e**–**f** The mitochondrial arrangement was in order, and swelling of mitochondria was significantly improved, the matrix was relatively uniform, the cristae was clear and the structure was complete in the QSG group. **g** Myocardial protein expressional levels of PGC-1α and UCP2 in HF model rats. A reduced level of PGC-1α was observed in the model group. After treatment with QSG, cardiac PGC-1α level was observably up-regulated. UCP2 expressional level was significantly increased following HF and downregulated with QSG treatment. n = 4 per group. Values are mean ± SE. Asterisks indicates significant differences. *P < 0.05, **P < 0.01

Transmission electron microscope photos of heart tissues were shown in Fig. 9. The mitochondria arranged orderly and densely, and were round or oval in the sham group (Fig. 9a). The mitochondrial membrane’s structure was complete, and the mitochondrial cristae and matrix are clearly visible and evenly arranged in the sham group (Fig. 9b). Compared to the sham group, mitochondrial arrangement was scattered, swelling was obvious, matrix was loose, and cristae was fuzzy with obvious partial fracture in the model group (Fig. 9c, d). Compared to the model group, mitochondrial arrangement was in order (Fig. 9e), and swelling of mitochondria was significantly improved, the matrix was relatively uniform, the cristae was clear and the structure was complete (Fig. 9f).

To further investigate the effect of QSG on mitochondrial function, the expressions of PGC-1α and UCP2 were examined. PGC-1α is a key nuclear receptor coactivator that can induce mitochondrial biogenesis [31]. A reduced level of PGC-1α was observed in the model group (*P* < 0.05, Fig. 9g). After treatment with QSG, cardiac PGC-1α level was significantly up-regulated in comparison to the model group (*P* < 0.05, Fig. 9g). UCP2, a mitochondrial anion carrier protein, can lead to the uncoupling of oxidative phosphorylation and a decline in ATP synthesis when it is activated [32]. The level of UCP2 was significantly increased in the model group compared to the sham group (*P* < 0.01), which could be downregulated by QSG (*P* < 0.01 versus the model group, Fig. 9g).

## Discussion

**Fig. 10:**
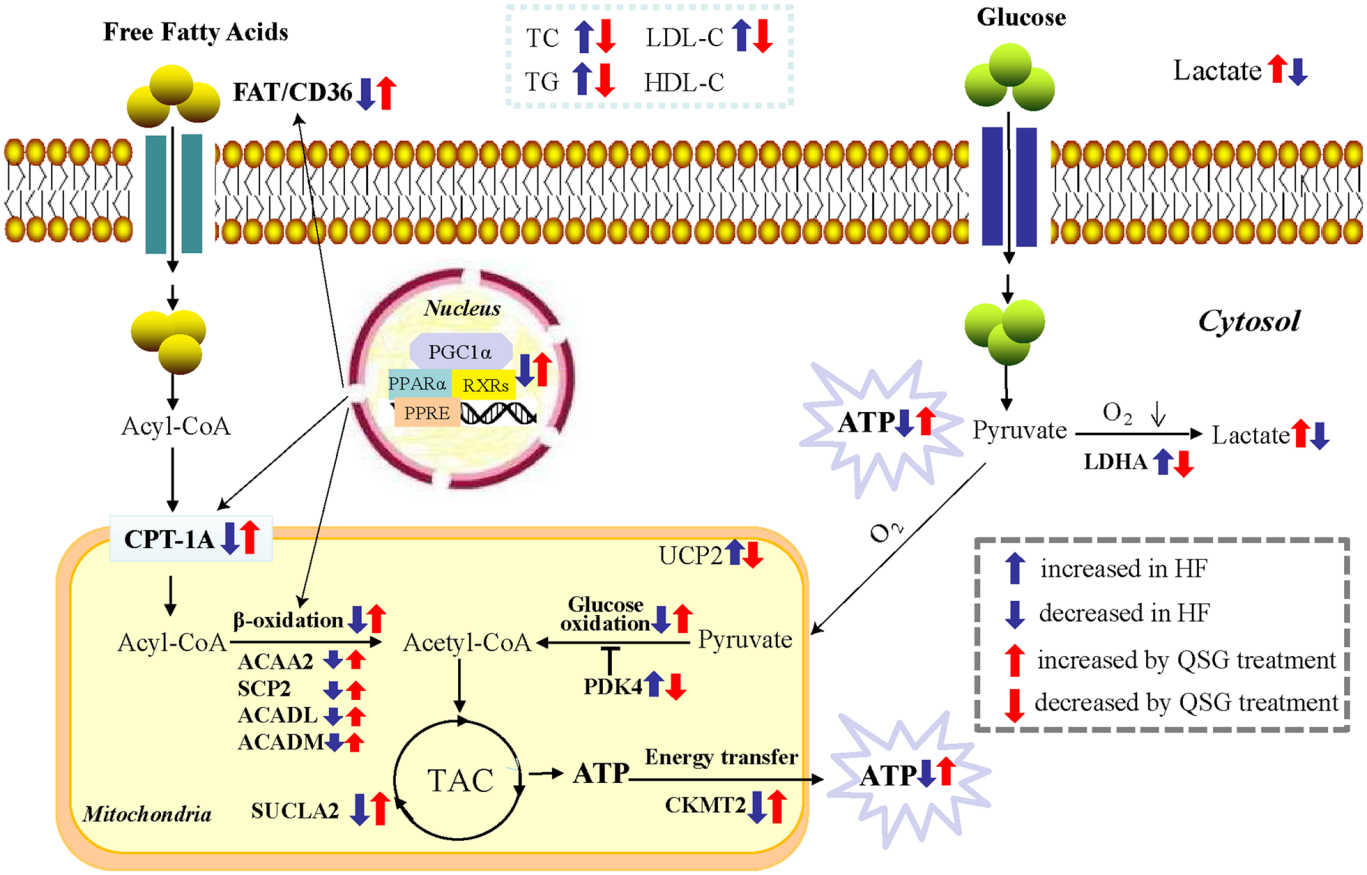
A schematic showing the effects of QSG on FA and glucose metabolism in HF induced by AMI. QSG exerted a remarkable regulatory effect on lipid metabolism by lowering serum levels of TC, TG and LDL-C. QSG activated FAT/CD36-CPT1-FAO signaling through upregulating the expressional levels of FAT/CD36, CPT1A, ACADL, ACADM, ACAA2 and SCP2, which would lead to an increase of FA uptake, transportation into mitochondria and β-oxidation. QSG promoted FA metabolism to a large extent on the up-regulation of transcriptional regulator PPARα, RXRα, RXRβ, RXRγ and PGC-1α. LDHA and PDK4 involved in glycolysis and glucose oxidation were all down-regulated by treatment with QSG, indicating QSG inhibited uncoupling of glycolysis from glucose oxidation. QSG facilitated TAC and the transfer of ATP from mitochondria to cytoplasm by increasing the protein levels of CKMT2 and SUCLA2. Moreover, the mitochondrial function was enhanced with QSG administration proved by the increased PGC-1α and the decreased UCP2

In this study, cine MRI and echocardiography were used to evaluate the effects of QSG on cardiac functions. Moreover, MRI is a non-invasive diagnostic tool applicable for simultaneous assessment of structural status of the heart during the cardiac cycles. Thus, the remodeling process and hemodynamic changes of the heart in HF rat model can also be captured accurately by MRI [33]. In this study, MRI and echocardiography results showed that QSG could improve cardiac functions and attenuate myocardial remodeling during development of HF. The cardiomyocytes were arranged in a disordered way and myocardial interstitial inflammatory cell infiltration could be observed in HF rats, accompanied with disorders of lipid metabolism. To further study the cardiac protective effect on energy metabolism, we made a comprehensive research on FA and glucose metabolism. As a result, we found that the protective effects of QSG were potentially mediated by regulating FA and glucose metabolism (Fig. 10).

### QSG promoted FA uptake, transportation into mitochondria and β-oxidation in HF

The FAT/CD36-CPT1-FAO pathway, mediating FA uptake, transportation into mitochondria and β-oxidation, was suppressed in HF model in our study, which was consistent with previous studies [9]. HF is consistently associated with decreased FA metabolism and therefore an approach to treat HF is to enhance FA metabolism [9]. QSG promoted the level of FAT/CD36 to increase FA uptake in cytoplasm of cardiomyocytes [25]. Furthermore, CPT1, which facilitates the transportation of long chain FA into the mitochondrial matrix [34], was also increased by treatment of QSG. ACADL, ACADM, ACAA2 and SCP2, the critical enzymes for β-oxidation of FA within the mitochondria [26–28, 35], were all up-regulated by QSG treatment, indicating that QSG could promote fatty acid β-oxidation.

### QSG activated PPARα-RXRs pathway to regulate the transcription of FA metabolism in HF

The protein level of PPARα, RXRα, RXRβ, RXRγ and PGC-1α in cardiac tissues of left ventricle in infarct border zone dropped significantly in the rat model of HF. The PPAR transcriptional complex controls the expression of FA utilization genes by binding to RXRs, and interacting with PGC-1α to recruit other cofactors to initiate gene transcription for FA metabolism [36, 37]. For instance, FA metabolism related molecules FAT/CD36, CPT1A, ACADL, ACADM and ACAA2 were all transcriptionally regulated by PPAR-RXRs signaling pathway [27]. There are three subtypes of PPAR, including PPARα, PPARβ and PPARγ. Among them, the principal transcriptional regulator of FA metabolism gene is PPARα, a member of the ligand-activated nuclear receptor superfamily [38, 39]. In addition, PGC-1α serves as a co-activator of PPARα and enhances FA oxidation and mitochondrial biogenesis through its synergistic effects with PPARα [31]. Our experimental study showed that PPARα-RXRs pathway was inhibited, which means that fatty acid metabolism was weakened in the rat model of HF. The up-regulated protein levels of PPARα, RXRα, RXRβ, RXRγ and PGC-1α in HF model rats indicate that QSG could promote FA metabolism by activation of PPAR-RXRs signaling pathway.

### QSG regulated abnormal glucose metabolism by inhibiting uncoupling of glycolysis from glucose oxidation in HF

The change of glucose metabolism was determined by PET-CT which can directly reflect the uptake of glucose located in myocardial tissue. Results showed that glucose was accumulated abnormally in periinfarct area in HF model group, whereas that in the sham group was intact. The abnormal increase of glucose in periinfarct area in the model group may be due to compensatory increasing glucose uptake from blood into the heart and local inflammatory response in the myocardium with low energy metabolism, which is consistent with the previous study [40]. The accumulating glucose was significantly metabolized after treatment with QSG.

To further study the mechanism of glucose metabolism in HF, We examined the key enzymes involved in glycolysis and glucose oxidation. QSG inhibited the final step of anaerobic glycolysis to reduce lactic acid production by downregulating the protein level of LDHA in HF model. Lactate level in serum and myocardial tissue was also confirmed to drop significantly with QSG treatment in this study. PDK4 decreases glucose oxidation by an inhibitory phosphorylation of the pyruvate dehydrogenase complex [29] and the expression of PDK4 was down-regulated with QSG administration, indicating that QSG could inhibit uncoupling of glycolysis from glucose oxidation.

### QSG facilitated TAC and the transfer of ATP from mitochondria to cytoplasm in HF

Tricarboxylic acid cycle and the transfer of ATP from mitochondria to cytoplasm are the key steps in energy metabolism. They are suppressed in the process of HF. SUCLA2 is a core enzyme of TAC [41] and the reduction of SUCLA2 in HF model rats would result in reduced ATP production [42]. CKMT2 which is responsible for the transfer of mitochondrial ATP to the cytoplasm [30] was also decreased in HF model rats in this study. In contrast, QSG facilitated TAC and the transfer of ATP from mitochondria to cytoplasm by increasing SUCLA2 and CKMT2 in HF rats.

### QSG protected mitochondrial function in HF

Mitochondria function is closely related to its structure, so first of all, the mitochondrial ultrastructure was observed with an electron microscope. The mitochondrial arrangement was scattered, swelling was obvious, matrix was loose, and cristae was fuzzy with obvious partial fracture in HF model rats. QSG treatment reduced the mitochondrial structural damage significantly. PGC-1α that controls mitochondrial function and biogenesis was up-regulated after treatment with QSG in HF. The expression of UCP2 was significantly increased in HF model, leading to a decline in ATP synthesis [32]. The decreased protein level of UCP2 in QSG-treated rats indicated that QSG could enhance mitochondrial function in cardiac cells of HF model.

QSG has been demonstrated to improve myocardial energy metabolism by regulating FA and glucose metabolism in the rat model of HF induced by AMI in this study. In our previous studies, we have established stable injury models of cardiomyocytes and fibroblasts in vitro [15, 43]. We will explore the regulatory mechanism of QSG on the energy metabolism of cardiomyocytes and myocardial fibroblasts, and endeavor to find out which compounds in QSG can regulate the targets of myocardial energy metabolism in the future study.

## Conclusion

The protective effects of QSG in treating HF were potentially mediated by improving FA metabolism,inhibiting uncoupling of glycolysis from glucose oxidation, facilitating tricarboxylic acid cycle, promoting the transportation of ATP from mitochondria to cytoplasm and restoring the mitochondrial function.

## Data Availability

The datasets used and/or analyzed during the current study are available from the corresponding author on reasonable request.

## Abbreviations

ACAA2: Acetyl-coenzyme A acyltransferase 2
ACADL: Acyl-coa dehydrogenase long chain
ACADM: Acyl-CoA dehydrogenase medium chain
AMI: Acute myocardial infarction
ATP: Adenosine triphosphate
CKMT2: Creatine kinase mitochondrial 2
CPT1: Carnitine palmitoyltransferase 1
EF: Ejection fraction
FA: Fatty acid
FAO: Fatty acid oxidation
FAT/CD36: Fatty acid translocase/cluster of differentiation 36
FDG: Fluorodeoxyglucose
FFAs: Free fatty acids
FOV: Field of view
FS: Fractional shortening
GAPDH: Glyceraldehyde 3-phosphate dehydrogenase
HDL-C: High-density lipoprotein cholesterol
HE: Hematoxylin-eosin
HF: Heart failure
LAD: Left anterior descending
LDHA: Lactate dehydrogenase A
LDL-C: Low-density lipoprotein cholesterol
LSD: Least significant differences
LV: Left ventricular
LVEDAWT: Left ventricular end-diastolic anterior wall thickness
LVEDD: Left ventricular end-diastolic diameter
LVEDV: Left ventricular end-diastolic volume
LVESAWT: Left ventricular end-systolic anterior wall thickness
LVESD: Left ventricular end-systolic diameter
LVESV: Left ventricular end-systolic volume
MRI: Magnetic resonance imaging
PET/CT: Positron emission tomography/computed tomography
PPARα: Peroxisome proliferator-activated receptor α
PGC1α: Peroxisome proliferator-activated receptor gamma coactivator 1-alpha
QSG: Qishen granule
RXRα: Retinoid X receptors α
RXRs: Retinoid X receptors
RXRβ: Retinoic X receptor β
RXRγ: Retinoic X receptor γ
SCP2: Sterol carrier protein 2
SE: Standard error
SUCLA2: Succinate-CoA ligase ADP-forming beta subunit
SUV: Standard uptake value
TC: Total cholesterol
TCA: Tricarboxylic acid
TCM: Traditional Chinese medicine
TG: Triglyceride
UCP2: Uncoupling protein 2
